# An immune-centric exploration of BRCA1 and BRCA2 germline mutation related breast and ovarian cancers

**DOI:** 10.1186/s12885-020-6605-1

**Published:** 2020-03-12

**Authors:** Ewa Przybytkowski, Thomas Davis, Abdelrahman Hosny, Julia Eismann, Ursula A. Matulonis, Gerburg M. Wulf, Sheida Nabavi

**Affiliations:** 1 Department of Computer Science and Engineering, University of Connecticut, Institute of System Genomics, Boston, MA USA; 2 Dana-Farber Cancer Institute, Boston, MA USA; 3 Beth Israel Deaconess Medical Center, Department of Hematology/Oncology, Harvard Medical School, Boston, MA USA

**Keywords:** BRCA1, BRCA2, Breast cancer, Ovarian cancer, Tumor mutation burden, Homologous recombination deficiency, Immunotherapy, Biomarkers, BRCAness, Platinum resistance, PARP

## Abstract

**Background:**

BRCA1/2 germline mutation related cancers are candidates for new immune therapeutic interventions. This study was a hypothesis generating exploration of genomic data collected at diagnosis for 19 patients. The prominent tumor mutation burden (TMB) in hereditary breast and ovarian cancers in this cohort was not correlated with high global immune activity in their microenvironments. More information is needed about the relationship between genomic instability, phenotypes and immune microenvironments of these hereditary tumors in order to find appropriate markers of immune activity and the most effective anticancer immune strategies.

**Methods:**

Mining and statistical analyses of the original DNA and RNA sequencing data and The Cancer Genome Atlas data were performed. To interpret the data, we have used published literature and web available resources such as Gene Ontology, The Cancer immunome Atlas and the Cancer Research Institute iAtlas.

**Results:**

We found that BRCA1/2 germline related breast and ovarian cancers do not represent a unique phenotypic identity, but they express a range of phenotypes similar to sporadic cancers. All breast and ovarian BRCA1/2 related tumors are characterized by high homologous recombination deficiency (HRD) and low aneuploidy. Interestingly, all sporadic high grade serous ovarian cancers (HGSOC) and most of the subtypes of triple negative breast cancers (TNBC) also express a high degree of HRD.

**Conclusions:**

TMB is not associated with the magnitude of the immune response in hereditary BRCA1/2 related breast and ovarian cancers or in sporadic TNBC and sporadic HGSOC. Hereditary tumors express phenotypes as heterogenous as sporadic tumors with various degree of “BRCAness” and various characteristics of the immune microenvironments. The subtyping criteria developed for sporadic tumors can be applied for the classification of hereditary tumors and possibly also characterization of their immune microenvironment. A high HRD score may be a good candidate biomarker for response to platinum, and potentially PARP-inhibition.

**Trial registration:**

Phase I Study of the Oral PI3kinase Inhibitor BKM120 or BYL719 and the Oral PARP Inhibitor Olaparib in Patients With Recurrent TNBC or HGSOC (NCT01623349), first posted on June 20, 2012. The design and the outcome of the clinical trial is not in the scope of this study.

## Background

The concept of cancer immunosurveillance, which claimed that the immune system can protect the host against the development of cancer, was proposed over 50 years ago by Burnet and Thomas [1, 2]. Recently, the evidence in favor of cancer immunosurveillance has been translated into new therapeutic approaches. DNA damage and genomic instability are closely linked to immunity. The production of tumor specific neoantigens is believed to be triggered by various mutations in the unstable cancer genome. Thus, immunosurveillance should be particularly relevant to BRCA1/2 germline mutation carriers, whose tumors have dysfunctional homologous recombination (HR), the main pathway for DNA double strand break repair [3].

The HR deficiency of hereditary breast and ovarian cancers makes them vulnerable to the inhibition of alternative pathways of DNA repair with inhibitors of Poly (ADP-Ribose) Polymerase (PARP) [4]. There are interests in expanding the use of PARP inhibitors to sporadic breast and ovarian cancers, some of which express phenotypes similar to hereditary tumors. For example, many sporadic TNBCs show deficiency in HR and demonstrate “BRCA-like” clinicopathological features, often referred to as “BRCAness” [5, 6]. “BRCAness” phenotype is also attributed to many hereditary and sporadic HGSOCs. However, the “BRCAness” phenotype is still poorly defined [7].

Due to having high TMB, BRCA1/2 germline mutation related tumors are considered to be candidates for immune checkpoint inhibition strategies, which were successful in highly mutated melanoma and lung cancers [8]. However, it has been shown that the BRCA1 gene product is a versatile regulator involved in many cellular functions in addition to its role in the DNA repair [9]. Moreover, the BRCA1 and BRCA2 gene products contribute in different ways to the tumorigenesis [10]. To find effective immune therapeutic strategies against hereditary breast and ovarian cancers, more information is needed about the relationship between genomic instability, phenotypes and immune microenvironments of those tumors.

The goal of this study was to explore genomic instability and phenotypes of hereditary and sporadic breast and ovarian cancers in relation to their immune microenvironments. Our results may help to find appropriate ways to stratify those tumors for testing various immune interventions. They will also help clarify the differences and similarities between BRCA1/2 germline mutation related phenotypes versus sporadic phenotypes of TNBC and HGSOC, and will help to define more precisely the elusive “BRCAness” phenotype.

## Methods

### Patients

The patients contributed to this study were selected for a clinical trial (#NCT01623349).

The genomic data was acquired from 19 patients out of total of 118 enrolled in the trial. Genetic material was extracted from Formalin-Fixed Paraffin-Embedded (FFPE) blocks prepared from tumors at diagnosis, before any treatment was administered to the patients. Eventually, all the 19 patients were treated heavily with conventional chemotherapy and fail the treatments. Details about the line of treatments are shown in Additional file 1: Table S1. This information may be relevant since it suggests that all the patients in this cohort could be considered resistant to conventional therapy. Design of a subsequent trial and the outcome of the trial are not in the scope of this hypothesis generating study and are available elsewhere (https://clinicaltrials.gov/ct2/show/NCT01623349).

The cohort was enriched in BRCA1/2 germline mutation carriers. The BRCA1/2 germline status was determined by a clinical test: MKS IMPACT™ tumor-profiling multiplex panel [11]. BRCA1/2 proteins were expressed in all samples, as determined at RNA level (data not shown)***.***

### RNA sequencing

RNA was extracted from Formalin-Fixed Paraffin-Embedded (FFPE) samples.

Qiagen RNeasy FFPE kit was used to extract RNA. TruSeq RNA and Access library prep kit was then used for preparing library for IIlumina RNA sequencing.

Illumina Sequencing: Illumina NextSeq 500 High Output v2 sequencer has been used to generate sequences in the FASTQ format. The 150-cycle kit for paired end 2 × 75 bp sequencing has been used with estimated 60 million total paired end raw reads per sample.

Sample extraction, library preparation and sequencing were done at the Center for Genome Innovation (CGI), Institute for System Genomics, University of Connecticut.

### RNA-seq data analysis

Quality Check: FASTQ file quality was checked using FASTQC v0.11.2. The summary reports showed no potential errors or warnings.

Alignment and Pre-processing: Reads were mapped using STAR Aligner tool v020201 to the human genome reference (hg19) downloaded from UCSC genome browser.

Transcripts quantification: Gene expression levels were obtained from the RNA-seq dataset using RSEM v1.2.31 with Ensembl gene annotation database.

Differential expression analysis: we have used EBSeq v1.21.0 for differential gene expression analysis of the RNA-seq data.

### Whole exome sequencing

FFPE samples were used for extracting DNA. Whole exome sequencing has been done at Memorial Sloan Kettering Cancer Center using Illumina sequencers. FASTQC v0.11.2 was used to check the quality of the paired end raw sequencing data in FASTQ format. The summary reports showed no potential errors or warnings.

Reads were aligned to hg19 genome reference using BWA v0.7.12-r1039 mem software tools.

### Subtyping breast and ovarian tumors

TNBC clinical trial samples were subtyped according to Lehmann et al. [12] into 6 subtypes, using their TNBC type tool run on genome-wide gene expression matrices for each sample [13], (http://cbc.mc.vanderbilt.edu/tnbc). Ovarian clinical trial samples were subtyped using the Classification of Ovarian Cancer (CLOVAR) scheme proposed by Verhaak et al. [14]. They defined a gene signature- set of 100 genes, used for classifying ovarian cancer into four subtypes. Single sample gene set enrichment analysis (SSGSEA) [15] was performed on each sample using these CLOVAR gene set. For every sample, SSGSEA outputs a score for each of the four subtypes. The highest score defines the classification for that sample. TNBC and CLOVAR subtypes for the The Cancer Genome Atlas (TCGA) dataset were downloaded from Lehmann et al. and Verhaak at al., respectively [14, 16]. Immune Subtyping on the clinical trial samples was performed using the Immune Subtype Classifier available from The Cancer Research Institutes iAtlas (https://www.cri-iatlas.org/about/). Immune Subtypes for TCGA data were download from iAtlas.

### Mutation burden analysis

Each patient’s tumor and normal BAM files were input into samtools v1.7 mpileup. Varscan somatic was called on each mpileup file yielding unfiltered vcf files. Varscan processSomatic was used to isolate high confidence SNV and indel calls, which were then false positive filtered using bam-readcount v0.8.0 and Varscan FPfilter. These high confidence, false positive filtered vcf files were used for analysis.

TCGA Mutation Annotation files for breast and ovarian cancer were downloaded from FireBrowse data version 2016_01_28 (firebrowse.org/).

### Leukocyte fraction and homologous recombination

Breast and Ovarian Leukocyte fraction and Homologous Recombination data was downloaded from iAtlas data portal (https://www.cri-iatlas.org/about/).

### Statistics

All statistical analysis was carrier out in R. Statistical significance was defined at a *p*-value < 0.05: **** < 0.0001, *** < 0.001, ** < 0.01, * < 0.05, measured by nonparametric Wilcoxon test, unless otherwise specified.

## Results

### Breast and ovarian cancers in BRCA1/2 germline mutation carriers show relatively low overall immune activity at diagnosis, compared to very immune active non-carriers

**Fig. 1:**
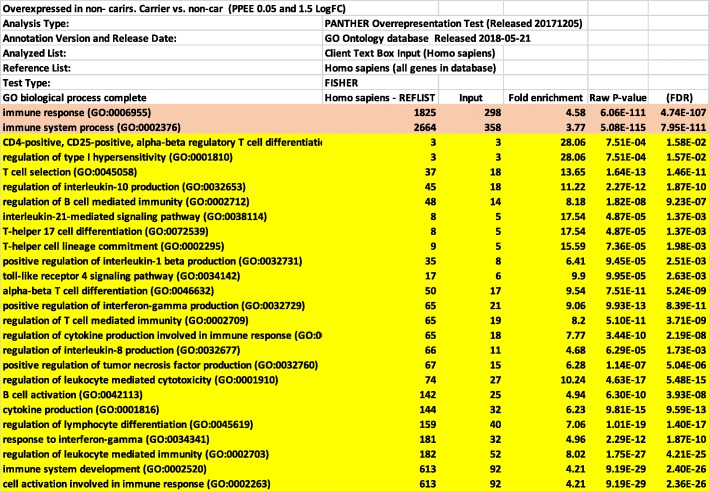
Biological processes enriched in breast and ovarian non-carriers from the clinical trial. The list of 813 genes was analyzed with Panther classification system (http://www.pantherdb.org). The table shows the top most significantly enriched biological process. The complete list of enriched processes is shown in Additional file 2: Table S2

In our clinical trial samples, we observed a striking difference in the gene expression profiles between germline mutation carriers and non-carriers. There were 1308 genes differentially expressed between carriers and non-carriers (Posterior Probability of equal expression < 0.05). Of these, 813 showed significantly higher expression in non-carriers (log fold change > 1.5) The biological processes most highly enriched in non-carriers identified with Gene ontology tool (Panther Classification System: http://www.pantherdb.org) were all related to immune functions (Fig. 1). Other biological processes which were also enriched in non-carriers include calcium ion transport and signaling, regulation of cell adhesion, motility and chemotaxis, protein secretion, cell signaling (MAPK, ERK1/2 and JNK), cell proliferation, differentiation and cell death (Additional file 2: Table S2). Many of these processes are related to biology of immune cells. Genes overexpressed in carriers, on the other hand, were not enriched for any particular biological process (data not shown).

**Fig. 2:**
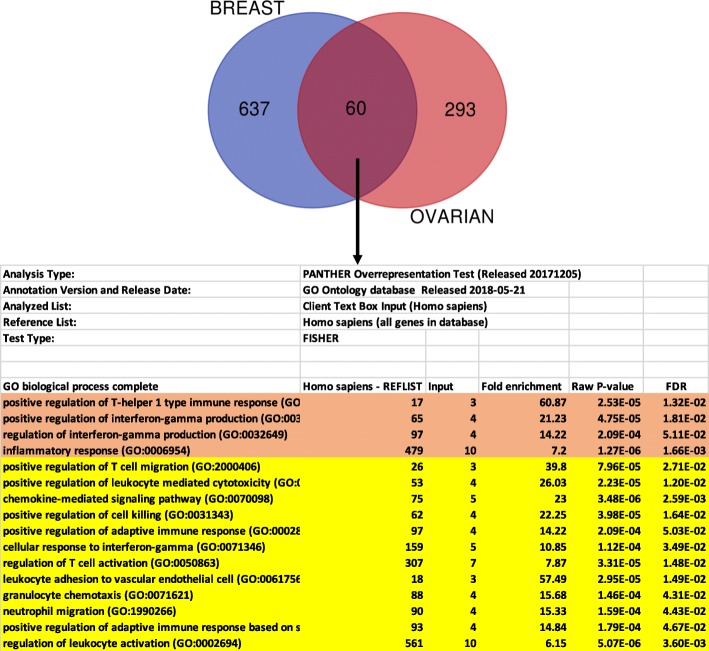
The common genes upregulated in breast and ovarian non-carriers from the clinical trial are involved in immune functions. 60 genes overexpressed in breast non-carriers overlapped with those overexpressed in ovarian non-carriers. The list of 60 genes was analyzed with Panther classification system (http://www.pantherdb.org). The table shows the top most significantly enriched biological process. The complete list of processes is shown in Additional file 7: Table S7

We have focused on the 500 biological processes, highly enriched in non-carriers, which were related to immune functions such as T cells differentiation and selection, B cells activation and regulation, production of various Interleukins and signaling via TNF alpha and interferon gamma. This data was highly significant suggesting that the immune environment of sporadic breast and ovarian cancers in our cohort was much more active relative to that of carriers of germline mutations in BRCA1/2 genes. This was independent from the type of germline mutation, BRCA1 or BRCA2 (Additional file 3: Table S3 and Additional file 4: Table S4) and was true for both types of cancers when analyzed independently (Additional file 5: Table S5 and Additional file 6: Table S6). Many genes overexpressed in breast non-carriers overlapped with those overexpressed in ovarian non-carriers (60 genes). The commonly upregulated genes in breast and ovarian non-carriers were all involved in immune functions (Fig. 2 and Additional file 7: Table S7).

**Fig. 3:**
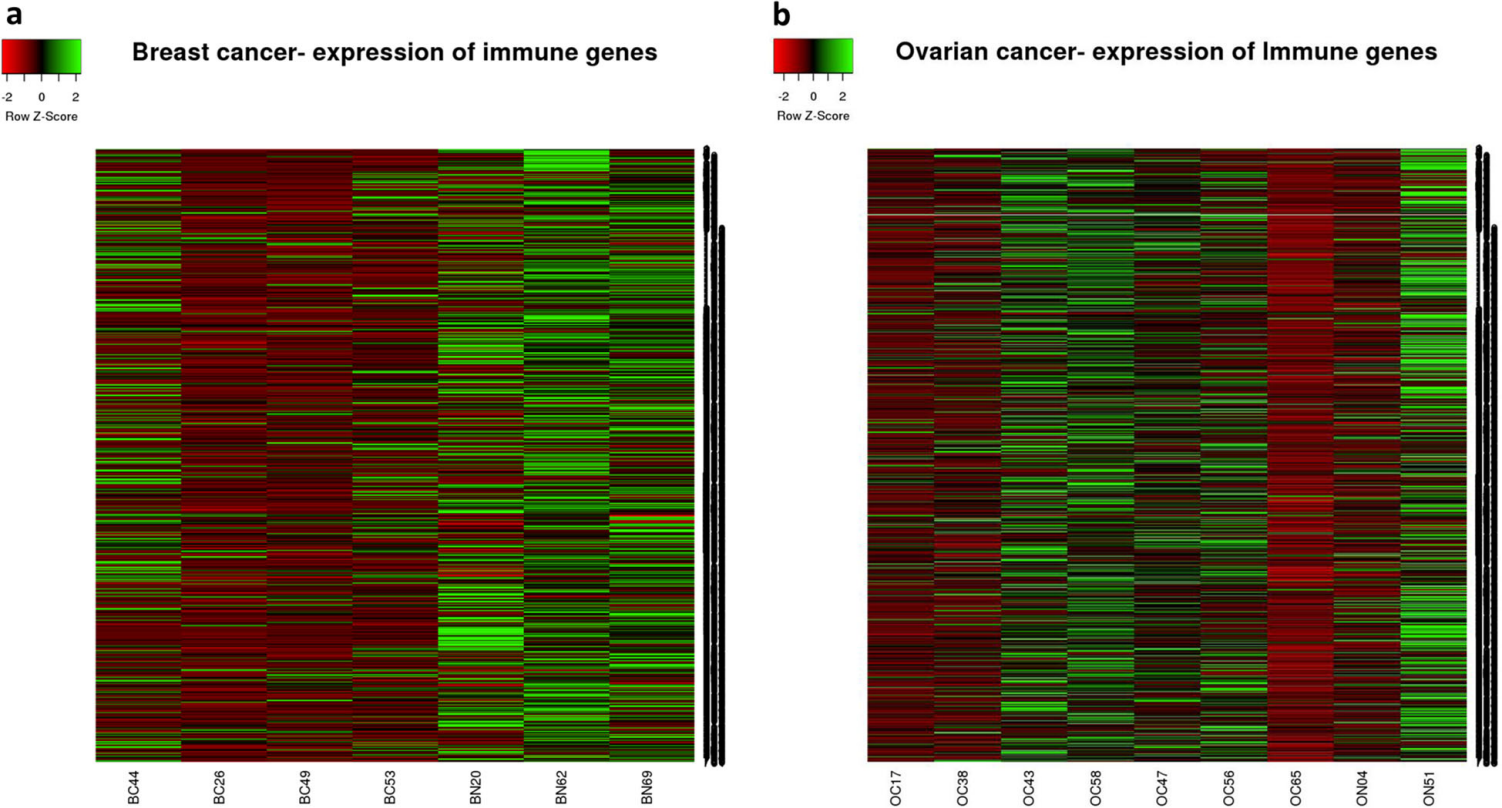
Patterns of expression of 782 genes representing 28 immune cell types, in samples from the clinical trial. Heat-maps represent expression of 782 genes in breast (**a**) and ovarian (**b**) samples from our cohort. The 782 gene list is shown in Additional file 8: Table S8. Breast carriers (BC), breast non-carriers (BN), ovarian carriers (OC), ovarian non-carriers (ON). The numbers correspond to the patient number (Fig. 4a)

Recently there have been attempts to characterize the immune components of the tumor microenvironment from high-throughput expression data [17–21]. The most complete analysis of immune infiltrates in tumor microenvironment was performed by the group of Trojanoski [21]. They developed a comprehensive and interactive database for immunogenomic studies: The Cancer Immunome Atlas (TCIA) (https://tcia.at/home), which allows exploration of specific immune related gene sets and assessment of cellular composition of infiltrates from 20 solid cancers. We have used their list of 782 genes, which characterize 28 different cell types present in tumor infiltrates [22] to analyze the global immune landscapes of individual carriers and non-carriers in our cohort (Fig. 3). The gene list is shown in Additional file 8: Table S8. All four breast carriers of germline BRCA1/2 mutation showed overall low expression of genes associated with various immune cell types, while three non-carriers showed relatively high expression of most of those genes. The picture was different for ovarian cancers, where some carriers and some non-carriers showed various expression of immune genes consistent with less robust differential expression results. Thus, the expression of 28 meta-gene sets validated our results obtained from differential expression analysis. Expression of these meta-gene sets can be a convenient way of representing global immune activity of tumors.

### BRCA1/2 germline mutation related breast and ovarian cancers show a range of phenotypes similar to that of sporadic cancers

There is still controversy if hereditary BRCA1/2 mutation related tumors represent a separate phenotypic identity. Both TNBC and HGSOC represent heterogenous groups of cancers and recently both tumor types were subdivided into several subtypes [12, 14, 16, 23–27]. Six subtypes of TNBC (IM, BL1, BL2, LAR, M and MSL) were identified from clustering of gene expression data [12]. The Immunomodulatory (IM) subtype is enriched in immune cell signaling. Two other subtypes (basal-like 1 and basal-like 2 (BL1 and BL2)) express high levels of the genes involved in cell proliferation and DNA damage response (DDR), however BL2 is of basal myoepithelial origin and can be distinguished by activated signaling pathways (EGF, NGF, MET, Wnt/β catenin and IGFR1) and glycolysis. Luminal androgen receptor (LAR) subtype is the most distinct of all subtypes, characterized by luminal features and expression of androgen receptor. Mesenchymal (M) and mesenchymal-stem like (MSL) subtypes are characterized by expression of genes involved in epithelial/mesenchymal transition. Patients with BL1 tumors show relatively good prognosis, while patients with BL2 tumors have very poor outcome [28].

Four subtypes of HGSOC (IMR, DIF, MES and PRO) were identified by gene expression profiling. The immunoreactive subtype (IMR) is enriched in immune cell signature, the differentiated subtype (DIF) expresses differentiation markers, the mesenchymal subtype (MES) is characterized by stromal expression signature indicating activated stroma, while the proliferative (PRO) subtype is characterized by low expression of ovarian cancer markers, but overexpression of proliferation and extracellular matrix (ECM) related genes. Importantly, the expression clusters distinguishing the subtypes strongly correlate with histological types of HGSOC [25]. Among all subtypes, the IMR shows the best prognosis and MES subtype has relatively poor outcome [14].

**Fig. 4:**
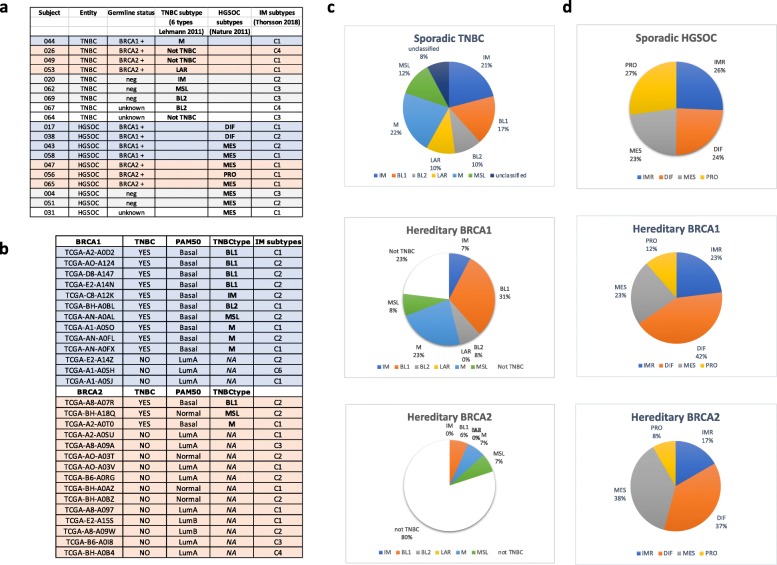
Subtypes of hereditary and sporadic breast and ovarian cancers in the clinical trial and in TCGA database. **a**) List of clinical trial samples. Subtyping of tumors from this cohort was obtained using TNBCtype tool (http://cbc.mc.vanderbilt.edu/tnbc) for breast cancers and CLOVAR scheme [14] for ovarian cancers. **b**) List of hereditary breast tumors from TCGA. Subtyping of these tumors was acquired from Lehmann at al [16]. **c**) Distribution of TNBC subtypes within TCGA breast cancers (sporadic TNBC and hereditary BRCA1 and BRCA2 related breast tumors). **d**) Distribution of HGSOC subtypes within TCGA ovarian cancers (sporadic HGSOC and hereditary BRCA1 and BRCA2 related ovarian tumors). The list of breast germline mutation carriers was established according the information acquired from CBioPortal (http://www.cbioportal.org) and iAtlas https://www.cri-iatlas.org/about/. The list of ovarian germline mutation carriers was established from CBioPortal (http://www.cbioportal.org) and it is shown in Additional file 9: Table S9. Immune Subtypes for our cohort were identified using tool available in iAtlas interactive platform and for TCGA samples were download from the site

Only one of six subtypes of TNBC (IM) and one of four subtypes of HGSOC (IMR) are characterized by a highly immune active microenvironment. We used a publicly available tool for TNBC classification developed by Lehmann to classify breast tumors from the clinical trial samples [13], ( http://cbc.mc.vanderbilt.edu/tnbc). The classification of HGSOC was obtained using the CLOVAR signature (see Methods section for details). Indeed, one of the three sporadic TNBC in this cohort was immunomodulatory, while two others belonged to different categories (MSL and BL2) (Fig. 4a). Breast tumors from BRCA1/2 germline mutation carriers expressed M and LAR subtypes and none were immunomodulatory. Interestingly, two BRCA2 germline mutation related breast tumors were classified as not TNBC. Most of the HGSOC from carriers and non-carriers of germline mutations belonged to MES subtype and none were immunomodulatory. Thus, none of the patients in this cohort, who carried germline mutation in BRCA1/2, developed highly immune-active tumors at diagnosis (Fig. 4a). In addition, none of the TNBC were classified as BL1, which is associated with good prognosis and the majority of HGSOC (70%) expressed MES subtype associated with the poor prognosis. This is consistent with the history of the patients in this cohort (lack of response to conventional therapies and progression to metastasis).

To put this data into perspective we examined the classification of all BRCA1/2 germline mutation related breast and ovarian tumors from The Cancer Genome Atlas (TCGA) datasets (Fig. 4b and Additional file 9: Table S9). Consistent with the subtyping in our clinical trial samples, few BRCA1 germline mutation related breast tumors in TCGA database are immunomodulatory (7% versus 21% of TNBC from non-carriers) and most BRCA2 germline mutation related breast cancers do not classify as TNBC (12 out of 15, 80%) (Fig. 4b and c). The results for ovarian cancers show a similar pattern. However, it is important to emphasize that HGSOC often express multiple signatures. Therefore, classification into mutually exclusive subtypes may be less specific than in other cancers [14]. Nevertheless, BRCA1 /2 germline mutation related HGSOC are not enriched in immunoreactive phenotype (Fig. 4d).

Thus, indeed BRCA1/2 germline mutation related tumors do not belong to the most immune active category of breast and ovarian cancers. The data also suggest that BRCA1/2 germline mutation related breast and ovarian cancers express range of phenotypes similar to sporadic cancers and therefore it is unlikely that they represent unique phenotypic identity within TNBC or HGSOC.

However, BRCA1/2 hereditary tumors have unique mutational signature [29] and BRCA 1 tumors have characteristic genomic copy number alterations [30]. Thus, it seems that mostly genotypes, but not phenotypes, make tumors related to BRCA1/2 germline mutation carriers unique.

### BRCA1/2 germline mutation related breast and ovarian cancers show relatively low overall immune activity in their microenvironment despite having elevated mutation burden

The relatively low immune activity in cancers (breast and ovarian) from BRCA1/2 germline mutation carriers is counterintuitive. Tumors with compromised DNA repair usually have a high mutational load and would be expected to generate a high number of neo-antigens [31]. In addition, hypermutated cancers such as melanoma or lung cancer, as well as colon cancer deficient in mismatch repair show positive response to immunotherapy [32–34].

**Fig. 5:**
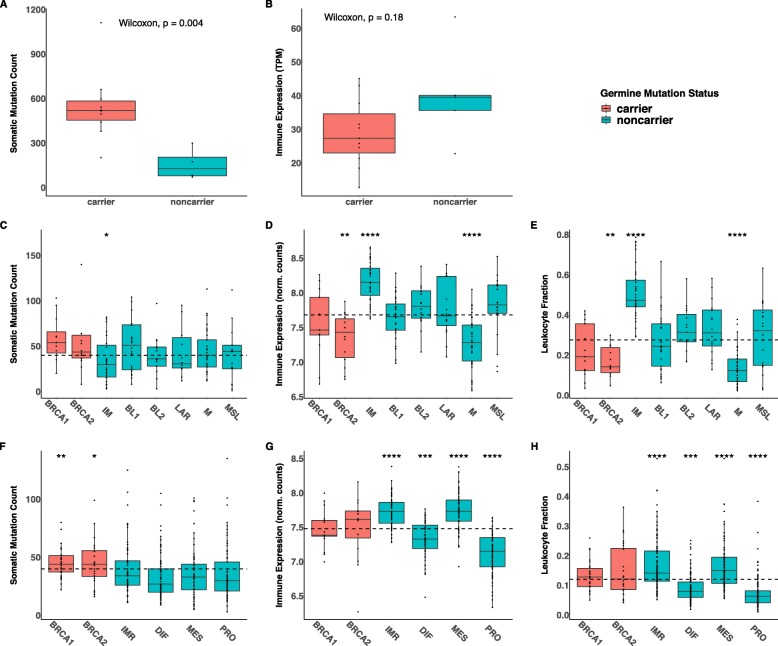
Hereditary breast and ovarian cancers from the clinical trial and from TCGA database show high TMB and low overall immune activity relative to the sporadic tumors. Data obtained for our cohort (**a**, **b**), data acquired for TCGA breast (**c**-**e**) and ovarian (**f**-**h**) cancers. **c** and **f**) Somatic mutation count acquired from CBioPortal (http://www.cbioportal.org), **d** and **g**) Global immune gene expression representing averaged expression of genes from 28 meta-gene sets. Expression data was downloaded from FireBrowse data version 2016_01_28 (this link http://firebrowse.org/) **e** and **h**) Leukocyte fraction acquired from Cancer Research Institute iAtlas https://www.cri-iatlas.org/about/. The dotted lines indicate the average value for all the samples in each panel

As expected, germline mutation carriers from our clinical trial samples show a higher tumor mutational burden (TMB) compared to non-carriers (Fig. 5a) and this is in contrast to global immune activity, which is lower in mutation carriers (Fig. 5b). Thus, we asked if there is a correlation between TMB and global immune activity in TCGA.

Within breast cancers, TMB was higher for BRCA1 and BRCA2 germline mutation carriers relative to non-carriers and was also elevated in BL1 subtype. Within HGSOC, TMB was higher only for germline mutation carriers and did not vary among other subtypes (Fig. 5c and f). Remarkably however, the global immune activity of tumor microenvironments, calculated as averaged expression of genes from 28 meta-gene sets, varied widely between subtypes (Fig. 5d and g).

Another measure of global immune activity is the leukocyte fraction of tumors. The leukocyte fraction for samples from TCGA is available on the web-based interactive platform: the Cancer Research Institute iAtlas https://www.cri-iatlas.org/about/. iAtlas was designed from extensive immunogenomic analysis and integration of the data for 33 cancer types [35]. The leukocyte fractions in subtypes of hereditary and sporadic TNBC and HGSOC from the TCGA database showed a very similar pattern to the expression of 28 meta-gene sets (Fig. 5e and h) and also did not correlate with TMB. Thus, BRCA 1/2 germline mutation related hereditary breast and ovarian tumors, have low overall immune activity within their tumor microenvironments despite their elevated TMB. The data suggest that diversity of immune responses in the microenvironments of hereditary and sporadic TNBC and HGSOC is likely determined by factors other than TMB.

### Pattern of genomic instability is different in BRCA1 versus BRCA2 germline related tumors

**Fig. 6:**
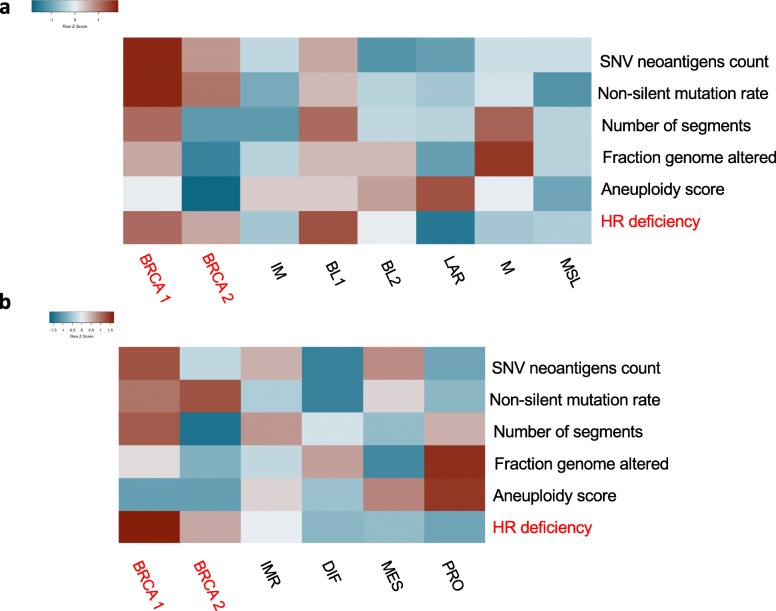
Pattern of genomic instability vary widely within hereditary and sporadic breast and ovarian cancers and it is different in BRCA1 versus BRCA2 germline related tumors. Heat-maps represent genomic instability measures in breast (**a**) and ovarian (**b**) cancers from TCGA. The data was acquired from Cancer Research Institute iAtlas (https://www.cri-iatlas.org/about/)

TNBC and HGSOC are characterized by frequent mutations in TP53 gene and a high degree of genomic instability. Considering that elevated TMB in hereditary breast and ovarian cancers was not associated with high immune activity in the tumor microenvironment, we looked at other measures of instability that potentially could influence immune response in breast and ovarian cancers. Recently, the extensive Pan-Cancer analysis of DNA damage repair (DDR) deficiencies in cancer was published [36] and the results were made available in iAtlas (https://www.cri-iatlas.org/about/). Using this resource, we explored several measures of genomic instability including: mutation load (expressed as non-silent mutation rate and SNV neoantigen count), CNV load (expressed as number of segments and fraction genome altered), aneuploidy and HR deficiency. Genomic instability varies widely between the subtypes of breast and ovarian cancers. As expected, all tumors from germline mutation carriers display high HR deficiency but also relatively low aneuploidy. Consistent with the results shown in Fig. 5, breast and ovarian cancers from germline mutation carriers have a relatively high mutation load compared to non-carriers. (Fig. 6a and b). BRCA2 related tumors reveal a very different pattern of instability compared to BRCA1 germline related tumors with a low CNV load. This confirms that the characteristic copy number pattern published earlier for hereditary breast cancers applies only to BRCA1-related tumors [30, 37]. The relationship between measures of genomic instability and the immune activity in tumors may be complex and require further investigation.

### High HR deficiency score characterize all BRCA1/2 germline mutation carriers and is predictive of response to platinum in HGSOC

**Fig. 7:**
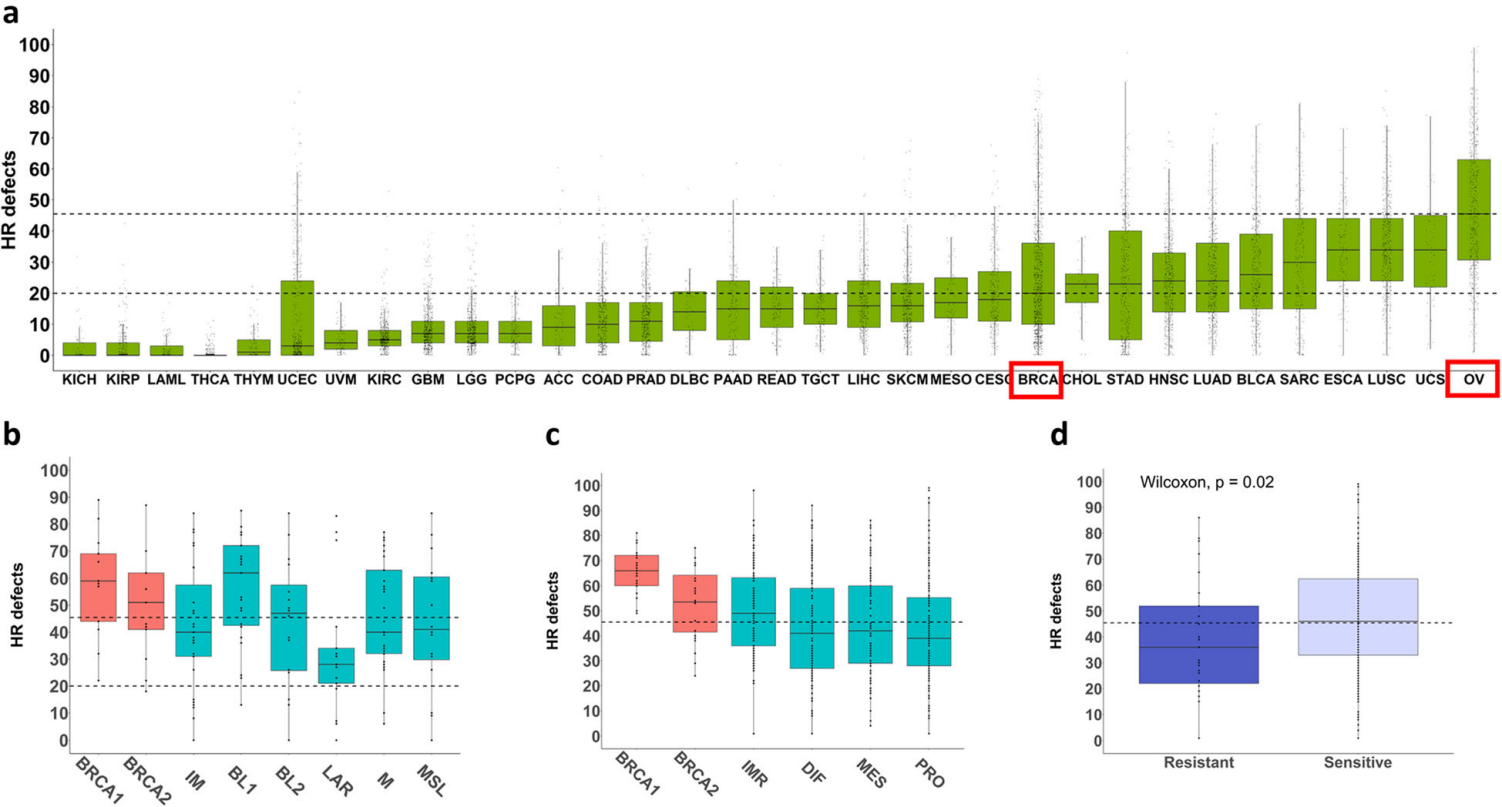
High HR deficiency score characterize most of TNBC and predicts platinum sensitivity in HGSOC. **a**) Distribution of HR deficiency score across 33 TCGA cancer types, **b**) across the breast cancer subtypes and **c**) across the HGSOC subtypes. The data was acquired from Cancer Research Institute iAtlas (https://www.cri-iatlas.org/about/). **d**) HR deficiency score in HGSOC, which are resistant or sensitive to platinum-based therapy. The sensitivity/resistance criteria were established according to Integrated genomic analysis of ovarian carcinoma [27] and applied to TCGA data (Additional file 10: Table S10). The dotted lines indicate mean HR deficiency score for all HGSOC (top line) and all breast cancers (bottom line)

HR deficiency is particularly relevant for hereditary TNBC and HGSOC. Ovarian cancer has the highest HR deficiency score of all 33 cancers included in TCGA (average value > 40) while breast cancers show much lower HR deficiency score (average value > 20) (Fig. 7a) [36]. However, TNBCs show a HR deficiency score as high as ovarian cancers (average value > 40), with the only exception of the LAR subtype (Fig. 7b). As expected, breast and ovarian tumors from BRCA1/2 germline mutation carriers have even higher HR deficiency scores (average value > 50 for BRCA2 and > 60 for BRCA1 mutation carriers) (Fig. 7b and c). Similar to TMB, HR deficiency did not correlate with immune activity. However, HR deficiency in ovarian cancers did correlate with platinum sensitivity (Fig. 7d). The sensitive and resistant ovarian cancers were selected from TCGA database. Tumors were defined as sensitive if there was no evidence of progression or recurrence at least 6 months from the date of primary platinum treatment. Tumors that recurred within 6 months of primary treatment were considered resistant [27]. The ovarian cancers sensitive to platinum had average HR deficiency score of 46.5 and resistant tumors had the score of 36.4. The difference was statistically significant.

### Distribution of “BRCAness” in subtypes of breast and ovarian cancers

The term “BRCAness” phenotype was coined to describe sporadic breast and ovarian cancers that behave like hereditary BRCA1/2-related tumors [5, 7].

The “BRCAness” characteristics of the subtypes of breast and HGSOC including BRCA1/2 germline mutation carriers from TCGA database are presented in Tables 1 and 2. The most important aspects of “BRCAness” phenotype chosen from literature were as follows: deficiency in HR, high genomic instability, frequent P53 mutations, but infrequent PI3K mutations in breast and ovarian cancers, in addition to basal like classification and high probability of pathological complete response (pCR) in breast cancers [6, 7, 28, 38, 39, 41, 42]. “BRCAness” is most often found in the BL1 and M subtypes of TNBC. Consistent with these results, most of the BRCA1 germline mutation carriers belong to BL1 or M subtype (Fig. 4c) and the “BRCA1-like” tumors selected according to copy number criteria also belong mostly to the BL1 and M category [30]. The LAR subtype, on the other hand, has frequent PIK3CA mutations and a low HR deficiency score. The IM subtype does not meet genomic instability criteria, MSL is not basal type and BL2 subtype is characterized by very low pCR. Importantly, BRCA2 germline related tumors do not express any attributes of “BRCAness” except high genomic instability.

**Table 1:**
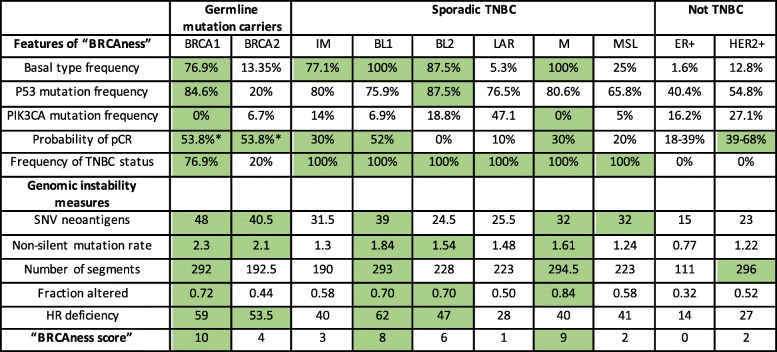
The “BRCAness” characteristics of breast tumors from TCGA database

The PAM50 status and TNBC status (absence of ER, PR and HER2) were taken from The Cancer Immunome Atlas (TCIA) https://tcia.at/home). The Lehmann subtyping was acquired from Lehmann at al [16].. P53 and PIK3CA mutation status were taken from cBioPortal (http://www.cbioportal.org)

Genomic instability measures represent median values calculated for each subtype using data on individual samples taken from iAtlas (https://www.cri-iatlas.org/about/). The values were considered positive for “BRCAness” (green cells) when they were equal or exceeded the threshold. The thresholds were as follows: 1). Frequencies of Basal type, P53 mutation, PIK3CA mutation and TNBC status expressed by BRCA1 germline mutation carriers 2). probability of pCR ≥30% and 3). The threshold for genomic instability measures represented the averaged value for all breast cancer types: 31.1 for SNV neoantigens, 1.54 for non-silent mutation rate, 234.3 for Number of segments, 0.59 for Fraction altered and 41.15 for HR recombination deficiency

*The pCR value given for BRCA2 germline mutation carriers applies to tumors expressing TNBC phenotype only [38] . The specific values for pCR vary depending on the type of therapy, but in most cases are higher for carriers of the germline mutations [39, 40]. pCR values for Lehmann subtypes were taken from Masuda at al. and Omarini at al. 2018 [28, 41]. pCR values for ER+ and HER2+ tumors were taken from I-SPY-2 TRIAL [42]

**Table 2:**
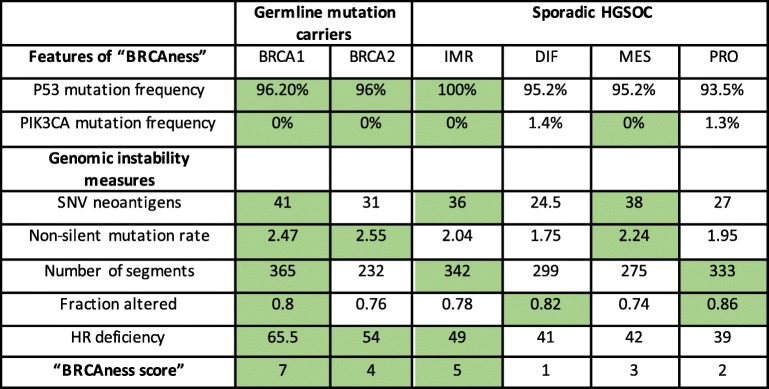
The “BRCAness” characteristic of ovarian tumors from TCGA database

HGSOC subtyping were taken from iAtlas (https://www.cri-iatlas.org/about/). P53 and PIK3CA mutation status were taken from cBioPortal (http://www.cbioportal.org)

Genomic instability measures represent median values calculated for each subtype using data on individual samples taken from iAtlas. The values were considered positive for “BRCAness” (green cells) when they were equal or exceeded the threshold. The thresholds for P53 and PIK3CA mutation status represent the frequencies expressed by BRCA1 germline mutation carriers. The threshold for genomic instability measures represent the averaged value for all HGSOC subtypes: 33 for SNV neoantigens, 2.2 for non-silent mutation rate, 307.7 for Number of segments, 0.8 for the Fraction altered and 48.4 for HR recombination deficiency

Similar analysis was performed for HGSOC subtypes (Table 2). According to our criteria, all subtypes of HGSOC score high on “BRCAness”.

### PD-L1 expression reflects overall magnitude of the immune response in breast and ovarian cancers

**Fig. 8:**
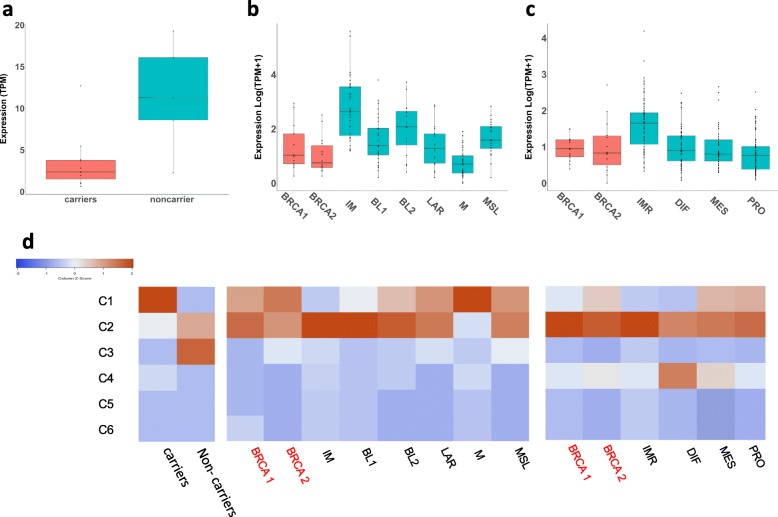
The immune response pattern in hereditary and sporadic breast and ovarian cancers from the clinical trial and from TCGA database. Expression of PD-L1 (CD274) in carriers and non-carriers from our cohort (**a**) in breast tumors from TCGA (**b**) and in HGSOC from TCGA (**c**). Heat-map showing relative contribution (percentage) of six universal intratumor immune states (C1-C6) within microenvironments of tumors from our cohort and from hereditary and sporadic breast and ovarian cancers form TCGA (**d**). PD-L1 for TCGA samples was downloaded from The Cancer Immunome Atlas (TCIA) (https://tcia.at/home). Immune Subtyping on the clinical trial samples was performed using the Immune Subtype Classifier available from The Cancer Research Institutes iAtlas (https://www.cri-iatlas.org/about/) and is also shown in Fig. 4a. Immune Subtypes for TCGA data were download from iAtlas

PD-L1 is the target for anti-PD-L1 antibodies, which are currently being examined in a phase II clinical trial (NCT02849496). PD-L1 RNA expression was significantly higher in samples from non-carriers of germline mutations compared to the carriers in our clinical trial samples (#NCT01623349) (Fig. 8a). Thus, higher overall immune activity corresponded with higher expression of this marker. We verified the expression of this marker in all subtypes of TNBC and HGSOC from TCGA database. All tumors expressed the protein, and the pattern of expression followed the pattern of overall immune activity in all samples including those from BRCA1/2 germline mutation carriers (see also Fig. 5c-h). The IMR subtype of HGSOC had the highest expression of PD-L1.

### The immune response patterns in TNBC and HGSOC

The immune landscape of 33 cancer types was recently published and made available on the web-based interactive platform [35], Cancer Research Institute iAtlas https://www.cri-iatlas.org/about/). They identified six universal intratumor immune states or response patterns. Briefly, C1, wound healing subtype, have elevated expression of angiogenic genes and high proliferative rate, C2, INF-γ subtype, have the highest M1/M2 macrophage polarization, C3 is an inflammatory subtype, C4 is lymphocytes depleted type displaying a more prominent macrophage signature, C5 is an immunologically quiet type and exhibit the lowest lymphocyte and highest macrophage response dominated by M2 and finally C6 is a TGF-β dominant type. When we applied the signatures for intratumor immune types (C1-C6) to our clinical trial samples, we found that the majority of non-carriers expressed C3 (inflammatory subtype), while majority of carriers expressed C1 (wound healing) subtype (Fig. 4a and Fig. 8d). The composition of the immune microenvironments within TNBC and HGSOC from TCGA varied widely, but almost universally the predominant subtypes were C2 (INF-γ and macrophage-enriched) and C1(wound healing). Some HGSOC expressed also C4 (lymphocytes depleted) subtype. Interestingly, two the most “BRCAness” expressing TNBC showed very different immune environments. BL1 tumors with higher overall immune activity relative to M tumors are predominantly (82.8%) associated with macrophage-enriched (C2) immune signature, while M tumors, which have overall very low immunoactivity, are predominantly (77.8%) associated with wound healing (C1) signature (Fig. 8d).

## Discussion

Cells that carry BRCA1/2 germline mutations have a high degree of genomic instability due to dysfunctional HR repair mechanisms and consequently a high TMB. High TMB has been associated with immunogenicity and response to immune checkpoint inhibitors such PD-1/PD-L1 antibodies in melanoma and lung cancer [8]. We found that breast and ovarian cancers from the clinical trial #NCT01623349, which carry BRCA1/2 germline mutation, as well as germline mutation carriers from TCGA database had a high TMB as reported previously [29, 43, 44] but did not score high on overall immune activity and PD-L1 expression relative to non-carriers. These data are consistent with recently published larger cohorts [45] who also reported no increase in PD-L1 expression or TILs in BRCA1-like tumors. However, it has to be kept in mind that the TMB in breast and ovarian cancers, even that from BRCA1 germline mutation carriers, is an order of magnitude lower compared to hypermutated cancers such as melanoma and lung cancer (The Cancer Immunome Atlas (TCIA) https://tcia.at/home). A lack of a positive correlation between TMB and immune infiltration in various types of tumors was recently described by others [46]. There are at least two possible caveats for the evaluation of TMB from genomic data. For one, the sequencing data is prone to inconsistencies due to various ways of processing NGS data and diverse criteria for relevant mutations [47]. Secondly, DNA sequencing data for highly immune infiltrated tumors can be affected by the immune cell “contamination”, reducing the readings for genomic instability.

The breast and ovarian cancers from BRCA1/2 germline mutation carriers show a unique responsiveness to PARP-inhibition and it was suggested that they express distinctive phenotype, which they share with some sporadic breast and ovarian cancers [7, 48]. Hence, the idea of “BRCAness”. We have addressed the extent of “BRCAness” phenotype in hereditary and sporadic TNBC and HGSOC from TCGA database. According to our results, “BRCAness” is not a unique phenotype of BRCA1/2 mutation carriers, but rather an attribute of majority of HGSOC and of a few subtypes of sporadic TNBC, which also happened to be the most frequent subtypes found within hereditary BRCA1 germline mutation carriers (BL1 and M). BRCA2 germline mutation related breast cancers on the other hand, do not express any “BRCAness” features except high genomic instability, and express the range of PAM50 phenotypes, similar to all sporadic breast cancers [40]. Consequently, the subtyping criteria developed for sporadic tumors can be applied for identifying “BRCAness” in sporadic and germline mutation associated tumors.

While BRCA1/2 related hereditary tumors may not have a unique phenotype, the breast cancers that carry BRCA1/2 deficiency have a unique genotype characterized by distinctive mutation profile [29, 30]. Distinct copy number signature (“BRCA1-like”) is also shared between cancers related to germline mutation in BRCA1 gene and sporadic cancers whose BRCA1 protein was inactivated through other mechanisms [49, 50]. Remarkably, the “BRCA1-like” subgroup distinguished with the copy number criteria had down regulated expression of proteins related to immune functions and was associated mostly with BL1 and M subtypes of Lehmann [30].

The global immune activity varies widely between breast and ovarian cancer subtypes and the immune microenvironments are heterogenous. Especially interesting finding is that two subtypes of TNBC (BL1 and M), which score high on “BRCAness” and are the most prevalent in hereditary BRCA1 germline mutation carriers have fundamentally different immune profiles, predominantly C2 and C1, respectively. The data suggest that the diversity of immune responses in the microenvironments of hereditary and sporadic TNBC and HGSOC may be associated with their particular phenotypes. In this respect, the subtyping of breast and ovarian tumors according to criteria developed for sporadic tumors may also be useful for testing various immune interventions. In our clinical trial samples, the majority of non-carriers expressed at diagnosis the C3 inflammatory immune profile, defined by elevated Th17 and Th1 genes, while majority of carriers expressed C1 wound healing profile. Further studies, with bigger cohorts, are needed to confirm this finding and to explore its significance.

In iAtlas, germline mutation carriers had the highest degree of HR deficiency of all breast and ovarian cancers. However, the surprising finding was that all ovarian cancers and almost all TNBC (with exception of LAR subtype), but not ER+ or HER2+ breast cancers, had a high degree of HR deficiency as well. This could explain the sensitivity of those tumors (TNBC and HGSOC) to DNA damaging chemotherapy and PARP inhibition. Indeed, we have shown that the platinum sensitive HGSOC from TCGA had significantly higher HR deficiency compared to resistant tumors. Similar results were obtained by Telli et al. Their study included both TNBC and ovarian cancers and used combined HR deficiency score, defined in a similar way as the HR score provided in iAtlas [51]. They suggested a clinical application of the score to identify TNBC (not deficient in BRCA1/2), which likely respond to platinum. If we apply their criteria for response, (HRD score ≥ 42), only two subtypes (BL1 and M) of sporadic TNBC and most of the subtypes of HGSOC will qualified as possibly sensitive to the therapy. Interestingly, the ovarian cancers from TCGA database, sensitive to platinum had HRD score above 42 (46.5) and resistant tumors had the score below 42 (36.4). Thus, our results validated the HR deficiency score as candidate biomarker for resistance to platinum.

BRCA1/2 germline related breast and ovarian cancers have the highest HR deficiency score of 33 tumor types analyzed in iAtlas and rather low mutational burden relative to hypermutated tumors (Cancer Research Institute iAtlas https://www.cri-iatlas.org/about/). We have found that they also show a low aneuploidy score relative to sporadic tumors. The low CNV load was also evident, but only in BRCA2 related breast and ovarian cancers. Genomic instability is the outcome of few processes going on simultaneously: DNA damage, DNA repair and immunoediting that is the clearance of cells with unrepaired damage by the immune system [52]. In BRCA1/2 germline mutation related tumors relatively low TMB (compared to hypermutated tumors) may be explained by effective immunoediting, rather than by effective DNA repair. It is tempting to speculate that immunoediting can compensate for the lack of adequate repair. If so, the immunoediting will have particular impact on hereditary tumors or sporadic tumors with BRCA1/2 dysfunction [53]. In support of this, low aneuploidy in BRCA1/2 germline related breast and ovarian cancers may also suggest more active immunosurveillence against cancer associated hyperploidy [54–56]. Active immunoediting in tumors from BRCA1/2 germline mutation carries may be consistent with a positive response to immune therapies in TNBC tumors despite the fact that they have relatively low TMB [57].

Looking from immune-centric point of view on patients with BRCA1/2 germline mutations, one wonders how BRCA1/2 deficiency influences the systemic immunity independent of its role in breast and ovarian tumorigenesis? Indeed, B-cell differentiation and maturation requires DDR [58] and BRCA1 protein have a direct role in B cells lymphomagenesis [59] suggesting the possible alterations in the systemic immunity of germline mutation carriers. In keeping with this, BRCA1/2 germline mutation carriers have higher risk of developing certain leukemias and lymphomas [60, 61].

The possibility of systemic immunity playing a role in tumorigenesis in the carriers of BRCA1/2 germline mutations was already suggested by others. It has been shown that DNA damage in cells that carry BRCA1/2 germline mutation was often overestimated and can hardly account for tumorigenesis [62]. Therefore, it was proposed that other factors, such as local inflammation and/or viral infections may put stress on immune system, which is already compromised by the germline mutation and promote the tumor formation in specific tissues such as breast and ovaries [63].

Cancer immunosuiveillence has been studied extensively for the last decade leading to the successful immune therapies [64]. Even though the immunotherapies are aimed at tumor microenvironment, systemic immunity is required for the process of tumor rejection after the therapy [65]. Thus, better understanding of the effects of cancer promoting hereditary mutations on the function of the systemic immunity may be very important. It can help to develop better immunotherapeutic strategies and new approaches for preventing and/or delaying the hereditary cancer, giving hope to many affected families.

## Conclusions

We have shown that TMB and other genomic instability measures are not associated with the magnitude of the immune response in hereditary BRCA1/2 related breast and ovarian cancers, as well as in sporadic TNBC and sporadic HGSOC. However, high HR deficiency score, characteristic to all ovarian cancers and most subtypes of TNBC may be associated with the sensitivity to platinum and potentially also PARP inhibition. Hereditary tumors express phenotypes as heterogenous as sporadic tumors with various degree of “BRCAness” and various characteristics of the immune microenvironment. The subtyping criteria developed for sporadic tumors can be applied for the classification of hereditary tumors and possibly also characterization of their immune microenvironment. Further studies are needed to clarify if immunoediting plays a particular role in protecting against accumulation of genetic damage in BRCA1/2 germline mutation carriers, and what is the impact of germline mutations on the systemic immunity.

## Supplementary information

**Additional file 1: Table S1.** Lines of treatment given to the patients, who were enroled into the trial (# NCT01623349) and whose expression profiles at diagnosis were analyzed in this study.

**Additional file 2: Table S2.** Panther analysis of genes overexpressed in non-carriers. All carriers vs. non-car (PPEE 0.05 and 1.5 LogFC).

**Additional file 3: Table S3.** Panther analysis of genes overexpressed in non-carriers. BRCA1 carriers versus non-carriers.

**Additional file 4: Table S4.** Panther analysis of genes overexpressed in non-carriers. BRCA2 carriers versus non-carriers.

**Additional file 5: Table S5.** Panther analysis of genes overexpressed in brest non-carriers. Breast carriers versus breast non-carriers.

**Additional file 6: Table S6**. Panther analysis of genes overexpressed in ovarian non-carriers. Ovarian carriers versus ovarian non-carriers.

**Additional file 7: Table S7.** Panther analysis of genes commonly overexpressed in breast and ovarian non-carriers.

**Additional file 8: Table S8.** Genes used for the analysis of the global immune activity of tumors.

**Additional file 9: Table S9.** Subtypes of hereditary and sporadic HGSOC from TCGA database.

**Additional file 10: Table S10.** HR deficiencies of platinum sensitive and resistant HGSOC from TCGA database.

**Additional file 11: Table S11.** The raw expression data for the cohort used in this study in TPM.

## Data Availability

The datasets analysed during the current study are available in CBioPortal (http://www.cbioportal.org), FireBrowse data version 2016_01_28 (http://firebrowse.org/), Cancer Research Institute iAtlas (https://www.cri-iatlas.org/about/), and The Cancer Immunome Atlas (TCIA) https://tcia.at/home). The raw expression data for the cohort used in this study in TPM are given in Additional file 11: Table S11. The RNA-seq raw data are available at the Gene Expression Omnibus under accession number GSE141142.

## Abbreviations

BL1: Basal like 1 subtype of TNBC
BL2: Basal-like 2 subtype of TNBC
BRCA1/2: Breast cancer type 1/2 susceptibility protein
CLOVAR: Classification of Ovarian Cancer
CNV: Copy number variation
DDR: DNA damage response
DIF: Differentiated subtype of ovarian cancer
ECM: Extracellular matrix
EGF: Epidermal growth factor
ER +: Estrogen-receptor-positive
ERK1/2: Extracellular signal–regulated kinases
FFPE: Formalin-Fixed Paraffin-Embedded
HER2 +: Human epidermal growth factor receptor 2 positive
HGSOC: High grade serous ovarian cancers
HR: Homologous recombination
HRD: Homologous recombination deficiency
IGFR1: Insulin-like growth factor 1
IMR: Immunoreactive subtype of ovarian cancer
INF-γ: Interferon gamma
JNK: The c-Jun N-terminal kinase
LAR: Luminal androgen receptor subtype of TNBC
M: Mesenchymal subtype of TNBC
MAPK: Mitogen-activated protein kinase
MES: Mesenchymal subtype of ovarian cancer
MET: A single pass tyrosine kinase receptor or hepatocyte growth factor receptor (HGFR)
MSL: Mesenchymal-stem like subtype of TNBC
NGF: Nerve growth factor
NGS: Next generation sequencing
PARP: Poly (ADP-ribose) polymerase
pCR: Pathological complete response
PD-1: Programmed cell death protein 1
PD-L1: Programmed death-ligand 1
PI3K: Phosphoinositide 3-kinases
PI3kinase: Phosphoinositide 3-kinase
PRO: Proliferative subtype of ovarian cancer
SNV: Single nucleotide variation
SSGSEA: Single sample gene set enrichment analysis
TCGA: The Cancer Genome Atlas
TCIA: The Cancer immunome Atlas
TGF-β: Transforming growth factor beta 1
Th1: T-helper cell 1
Th17: T-helper cell 17
Tils: Tumor infiltrating lymphocytes
TMB: Tumor mutation burden
TNBC: Triple negative breast cancer
TNF: Tumor necrosis factor
Wnt/β catenin: The canonical Wnt pathway
